# The Mobile Phone Addiction and Depression Among High School Students: The Roles of Cyberbullying Victimization, Perpetration, and Gender

**DOI:** 10.3389/fpsyg.2022.845355

**Published:** 2022-04-27

**Authors:** Wenzhi Wu, Yongchuan Chen, Xiuying Shi, Hua Lv, Rui Bai, Zhichao Guo, Lei Yu, Yilin Liu, Jianping Liu, Yatang Chen, Yong Zeng

**Affiliations:** ^1^The Sixth Affiliated Hospital of Kunming Medical University, Yuxi, China; ^2^Yuxi Nationalities High School, Yuxi, China; ^3^The Second Affiliated Hospital of Kunming Medical University, Kunming, China; ^4^Yuxi Third Middle School, Yuxi, China

**Keywords:** mobile phone addiction, depression, cyberbullying victimization, cyberbullying perpetration, high school students

## Abstract

**Objective:**

To examine the relation between mobile phone addiction and high school students’ depression, and its inner mechanism—the sequential mediating roles of the cyberbullying victimization and the cyberbullying perpetration in this relationship.

**Methods:**

1297 high school students were recruited to complete the Smartphone Addiction Scale, European Cyberbullying Intervention Project Questionnaire and the Center for Epidemiological Studies Depression Scale.

**Results:**

(1) Mobile phone addiction was positively correlated with and high school students’ depression; (2) cyberbullying victimization and the cyberbullying perpetration significantly mediated the relation between mobile phone addiction and high school students’ depression, which contained tow mediating paths—the independent mediating effects of cyberbullying victimization and the sequential mediating effect of cyberbullying victimization and the cyberbullying perpetration; (3) there are gender differences in the sequential mediation model, and boys who are victims of cyberbullying are more likely to develop into cyberbullying perpetrators than girls.

**Conclusion:**

The results of this study indicate that depression among high school students with mobile phone addiction can be eliminated through the development of cyberbullying victimization and the cyberbullying perpetration.

## Introduction

Mobile phone addiction is a new manifestation of Internet addiction in the mobile era. It refers to a behavioral addiction in which users have psychological and behavioral problems due to the abuse of mobile phones (Su et al., 2014). Depression is a common negative emotional experience that is harmful to an individual’s mental health and, in severe cases, may increase the likelihood of suicide in the individual (Maalouf et al., 2011). With the development of mobile Internet, the relationship between mobile phone addiction and depression has been paid more and more attention by researchers. Existing findings suggest a significant positive correlation between mobile phone addiction and depression (Çağan et al., 2014; Xiaodan et al., 2016; Alhassan et al., 2018). Teenagers who spend longer on social media with mobile phones score higher on the depression scale on average (Calpbinici and Tas Arslan, 2019), and retrospective studies have also shown that mobile phone addiction can lead to depression, anxiety, and sleep problems (Thomee, 2018).

On the other hand, the study suggests that adolescents with problematics mobile phone use are more exposed to cyberbullying (Mendez et al., 2020; Sheinov, 2021; Smale et al., 2021). Cyberbullying refers to aggressive and hostile attacks by a group or individual using electronic media, especially mobile phones or the Internet, repeatedly and permanently against victims who cannot easily protect themselves (Smith et al., 2008; Lim, 2013; Kowalski et al., 2014; Costa et al., 2019). Studies have shown that 14 to 49% of students said they have had cyberbullying behavior (Låftman et al., 2013; Li et al., 2018; Calpbinici and Tas Arslan, 2019), and this proportion is increasing; and the percentage of respondents who have experienced cyberbullying incidents ranges from 10 to 42% (Kraft, 2006; Lucas-Molina et al., 2018; Sánchez Domínguez and Magaña Raymundo, 2018; Cañas et al., 2019). Studies have confirmed the significant effects of mobile phone addiction and cyberbullying on depression in adolescents (Calpbinici and Tas Arslan, 2019; Cañas et al., 2019; Iranzo et al., 2019; Liu et al., 2020a), but the specific mechanism of action between the three has yet to be explored. Therefore, the present study aims to examine the mechanism of mobile phone addiction and cyberbullying in the development of depression in high school students, thus providing important research support for campus bullying prevention and adolescent mental health interventions in the context of the Internet age.

### High School Students’ Mobile Phone Addiction Can Lead to Depression

Numerous studies have found that addiction and depression often go hand in hand, and that addiction increases the risk of depression and is an important predictor of depression (Oh et al., 2013). A 4-year follow-up study confirms that Internet addiction is a stable cause of depression (Joseph et al., 2016). Therefore, in the development of depression, Internet addiction is considered to be a significant risk factor. In recent years, with the rapid development of mobile Internet, researchers have found that mobile phone addiction, as another important aspect of behavioral addiction after Internet addiction, is also significantly positively correlated with depression (Alhassan et al., 2018), which has potential effects on individual depression (Jun, 2016; Yang C. et al., 2019). Studies have examined the intermediation of emotional and cognitive factors between mobile phone addiction and depression (Li et al., 2017; Yang X. et al., 2019), but less attention to the indirect effects that mobile phone addiction may trigger related online behaviors such as cyberbullying. Previous studies have focused on mobile phone use and cyberbullying among college students (Bailin et al., 2014), and with the development of information society and economy, mobile phones have become more common among high school students, facilitating their lives while increasing their risk of cyberbullying (Mendez et al., 2020; Smale et al., 2021; Yadav and Chakraborty, 2021).

Based on these previous findings, the present study proposes hypothesis H1: High school students’ mobile phone addiction can lead to depression. That is, high school students addicted to mobile phones have a higher level of depression.

### Mobile Phone Addiction Increases the Risk of Cyberbullying Among High School Students

Mobile phone addiction is characterized by Inability to control craving, Feeling anxious and lost, Withdrawal/escape and Productivity loss (Leung, 2008). The study found that phone and Internet use for too long caused by Inability to control craving was a risk factor for cyberbullying (Kim et al., 2013; Alvarez-Garcia et al., 2015; HwaJin and Wanju, 2021). The Productivity loss of mobile phone addiction can lead to depression, anxiety, stress, sleep, and health problems in individuals, and increase the risk of individual cyberbullying (Sheinov, 2021). Social media addiction caused by Withdrawal/escape can also cause users to experience cyberbullying (Jain and Agrawal, 2020). Moreover, the compulsive examination of cell phones and cellphone vibration hallucinations caused by Feeling anxious and lost are related to the victimization and implementation of cyberbullying (Catone et al., 2020). Kowalski uses the General Aggressive Model to explain cyberbullying (Kowalski et al., 2014). GAM was based on cognitive knowledge structures (i.e., scripts and patterns) and integrating the development of cyberbullying through three parts: the input of individual and contextual factors, cognitive, emotional, and wake-up pathways that affect current internal states, and the assessment and decision-making processes that lead to resulting behaviors (Anderson and Bushman, 2002). Results from inputs enter the assessment and decision-making process through their impact on cognition, emotion, and wakefulness, both to determine the near-end process (focusing on the assessment and decision-making process in the context of cyberbullying) and to long-term negative outcomes for adolescents (e.g., depression, anxiety, behavioral problems, etc.) (Liu et al., 2020b,^2021^). These long-term negative behaviors and psychological outcomes can occur if individuals are involved in cyberbullying for long periods of time as victims or perpetrators. Technology use, such as mobile phone addiction, Internet addiction, etc., as personal factors, is directly related to cyberbullying behavior (Gül et al., 2018; Chung and Shin, 2020). The high level of bullying victims is significantly associated with high levels of problem internet use and mobile phone addiction (Li et al., 2020), while the victims develop higher levels of depression, perceived stress, loneliness, and social anxiety (Buelga et al., 2012; Iranzo et al., 2019; Bochkareva and Strenin, 2021), low self-concept, life satisfaction and emotional intelligence (Cañas et al., 2019), leading to suicidal tendencies, aggression, alcohol and drug abuse, truancy, and poor grades (Kang, 2015; Tözun, 2018). Therefore, the effect of mobile phone addiction on high school students may be achieved by increasing the risk of cyberbullying victimization.

Since there is no research to examine the combined effects of mobile phone addiction and high school students’ cyberbullying victimization on depression, the present study proposes hypothesis H2: Mobile phone addiction of high school students is positively correlated with cyberbullying victimization, which in turn is positively correlated with depression. In other words, high school students cyberbullying victims mediates the link between mobile phone addiction and depression.

### Mobile Phone Addiction Can Inspire High School Students to Commit Cyberbullying

Studies on Internet addiction have found that Internet addiction is associated with an increase in cyberbullying and bullying among adolescents (Floros et al., 2013), and that the rate of cyberbullying victimization and the prevalence of cyberbullying in the Internet addiction group are significantly higher than in the non-addiction group (Chang et al., 2015). On the one hand, individuals who spend more time on the Internet learn more about the use of technology, creating a power imbalance between bullying perpetrators and victims, then the cyberbullying intentions are more likely to develop into behavior (Shaikh et al., 2021); On the other hand, research shows that there is a positive correlation between internet usage time and cyberbullying perpetration (Tsimtsiou et al., 2017; Chacon-Borrego et al., 2018). Junior school students who spend more time playing games on weekdays are more likely to be involved in cyberbullying (HwaJin and Wanju, 2021), and younger students who are more active in using mobile phones are more likely to engage in cyberbullying than other students (Shin and Ahn, 2015). What’s more, there is a significant positive correlation between the factors in the Teen Smartphone Addiction Scale and the Cyberbullying Injury Scale (Chung and Shin, 2020). Teenagers who perpetrate cyberbullying score higher on depression, anxiety, negative self-esteem, physicalizing, and hostility (Calpbinici and Tas Arslan, 2019), so high school students with technology use problems such as mobile phone addiction are more likely to develop higher levels of cyberbullying, leading to depression (Yuan and Liu, 2021).

Since there is no research to confirm the positive predictive effect of cell phone addiction on cyberbullying among high school students, the present study proposes hypothesis H3: Mobile addiction of high school students is positively correlated with cyberbullying perpetration, which in turn is positively correlated with depression. In other words, high school students cyberbullying perpetration mediates the link between mobile phone addiction and depression.

### Victims of Cyberbullying Are More Likely to Develop Into Cyberbullying Perpetrators

Research shows that adolescent cyberbullying victimization is a predictor of cyberbullying (Álvarez-García et al., 2018; Ramos Salazar, 2021), while children who experience cyberbullying exhibit more aggressive behavior (Ijachi, 2019). GAM puts forward the path of how cyber victims can become cyber bullies. As a stressful event, cyber bullying experience consumes victims’ limited psychological resources. When completing tasks that require self-control such as experiencing the cyberbullying, the psychological resource will gradually be consumed, leading to a continuous decline in subsequent self-control performance (Baumeister et al., 2007), and more inclined to react impulsively, such as cyberbullying. In addition, the Frustration-Attack Hypothesis states that an individual’s frustration can lead to a “state of readiness” for attack behavior that can also be caused by other’s attacks and the habit of attack that has been developed (Berkowitz et al., 2016). Thus, victims of cyberbullying are more likely to develop an intention to bully others, that is, to gain dominance in a bullying way to show their strength, thereby feeling rejection from peers, and to have negative feelings such as depression in the bullying group (Reijntjes et al., 2010).

Since there is currently no direct evidence to support the positive projections of cyberbullying victimization on perpetration among high school students, the present study suggests hypothesis H4: Cyberbullying victimization is positively correlated with perpetration. Thus, the association between mobile phone addiction and depression is sequentially mediated by cyberbullying victimization and perpetration.

### Gender Differences in Cyberbullying Behavior of High School Students

In online behavior, gender differences are an unavoidable topic. Related meta-analysis believes that gender is the influencing factor of cyberbullying (Kowalski et al., 2014), but whether it is a male or a female is more likely to become the perpetrator, the research has no consistent results (Beckman et al., 2013; Lapidot-Lefler and Dolev-Cohen, 2015). Most studies have found that women are more likely to become victims of cyber bullying than men (Moreno–Ruiz et al., 2019). Girls prefer to use social networking sites and use multiple social media tools at the same time, so they may be more vulnerable to cyberbullying victimization (Messias et al., 2014). In addition, girls with poor interpersonal relationships are more likely to suffer cyberbullying (Jing et al., 2009). However, surveys have shown that boys are more vulnerable to bullying (Erdur-Baker, 2010). After being victimized by cyberbullying, boys’ adventurous and impulsive personality traits may be more involved in violence and show higher levels of cyberbullying perpetration (Aricak, 2009; Gonzalez-Cabrera et al., 2019; Martinez-Pecino and Duran, 2019). Research suggests that boys are more prone to cyberbullying, which may be related to their personality and online preferences. Boys are more impulsive and like to play adversarial online games, while girls are more tolerant and less exposed to adversarial situations (Baldry et al., 2016; Tolga et al., 2017). Wright believes that the occurrence of cyberbullying has nothing to do with gender, and that those with masculine tendencies are relatively more likely to develop cyberbullying (Wright, 2017). At the same time, the personality characteristics of boys who are not good at expressing their emotions can also lead to the appearance of negative emotions such as depression (Pečjak and Pirc, 2017; Cañas et al., 2019). Therefore, boys who have suffered cyberbullying may be more likely to use the same method to conduct cyberbullying on others than girls.

However, due to the inconsistent conclusions on gender differences in cyberbullying behavior in existing studies, the above relationship still lacks empirical support. Based on this, the present study proposes Hypothesis H5: High school students have gender differences in cyberbullying behavior, and male groups are more likely to develop from cyberbullying victimization to cyberbullying perpetration, that is, there are gender differences in the sequential mediation of cyberbullying victimization and cyberbullying preparation between high school students’ mobile phone addiction and depression.

**FIGURE 1:**
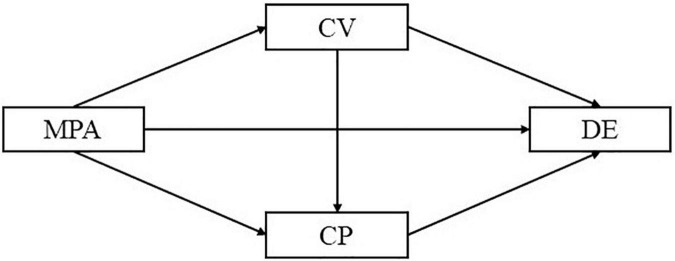
A hypothetical sequential mediating model diagram. MPA, mobile phone addiction; CV, cyberbullying victimization; CP, cyberbullying perpetration; DE, depression.

Cyberbullying victims caused by mobile phone addiction can cause depression in high school students, and victims tend to develop into cyberbullying practitioners, further increasing the level of depression. A multiple mediation model (Figure 1) was established for this study in order to test all possible mechanisms of these two mediators.

## Materials and Methods

### Participants

A cluster sampling method was adopted to select first-year students from two senior middle schools in a prefecture-level city in southwestern China as the survey participants in November 2021. Subject to the consent of the class teacher and the students themselves, postgraduates in psychology and psychiatry who had undergone uniform training would conduct group tests as a unit of the class. The participants were required to complete the questionnaire after reading the instruction carefully, and the test will take about 20 mins. A total of 1,400 questionnaires were distributed in the study. Questionnaires with the same answers or obvious answering patterns and more than 15% or more unanswered questions in any scale were excluded. Finally, 1,297 valid questionnaires were obtained, with an effective rate of 92.643%. Among them, boys accounted for 38.9% and girls accounted for 61.1%; urban students accounted for 25.7%, and rural students accounted for 74.3%; the average age was 15.50 years (*SD* = 0.573 years).

The questionnaire and methodology for this study was approved by the Human Research Ethics committee of the Kunming Medical University (Ethics approval number: 2021kmykdx6f66).

### Measurements

#### Smartphone Addiction Scale

The Smartphone Addiction Scale compiled by Su et al. (2014) has a total of 22 items, including withdrawal behavior (referring to a negative psychological or behavioral reaction when not participating in mobile phone activities, e.g., “If I can’t use a mobile phone for a period of time, I will feel anxious.”), salience behavior (referring to the use of smartphones that occupies thinking and behavioral activities, e.g., “I feel the need to spend more time on my mobile phone to be satisfied.”), social comfort (referring to the role of smartphone use in interpersonal communication, e.g., “I would rather choose a mobile phone for chatting than face-to-face communication.”), negative effects (referring to the decline in work and study efficiency due to the excessive use of smartphones, e.g., “Procrastination caused by playing with smartphones has brought me a lot of trouble.”), and use of application (App) (referring to excessive use of smartphone applications, e.g., “I will open some mobile applications unconsciously.”) and renewal of App (referring to the excessive attention of smartphone users to application updates, e.g., “I will be concerned about the recent new app and download it to my phone.”) six dimensions (Su et al., 2014). The scale uses Likert five-point scoring, 0 means “very inconsistent,” four means “very consistent.” The average score of all topics represents the teenager’s degree of mobile phone addiction, and the higher score means the higher the level of addiction. Previous studies have shown that the scale has good reliability and validity. The Cronbach’α coefficient of the scale in this study is 0.900. The confirmatory factor analysis shows that: χ^2^*/df* = 6.835, *RMSEA* = 0.065, *SRMR* = 0.053, *TLI* = 0.912, *CFI* = 0.933, indicating that the scale has good reliability and validity.

#### European Cyberbullying Intervention Project Questionnaire

The Chinese version of the European Cyberbullying Intervention Project Questionnaire revised by Zhu et al. was used. The original scale has a total of 22 items, and after translation and adjustment, it contains a total of 14 items, including two dimensions, which measure cyberbullying victimization (e.g., “Someone sent me threatening or harassing messages via SMS or social media.”) and cyberbullying perpetration (e.g., “I have published inflammatory rumors that have damaged others’ reputations.”), respectively. This scale has been validated locally among Chinese adolescents, showing good validity and reliability (Zhu et al., 2021). This scale uses Likert five-point scoring, 0 means “never,” 1 means “occasionally,” 2 means “sometimes,” 3 means “often,” 4 means “almost every day.” The Cronbach’α coefficient of the cyberbullying victimization scale in this study is 0.782, and the cyberbullying perpetration scale is 0.767, indicating that the scale has good reliability.

#### The Center for Epidemiological Studies Depression Scale

The Center for Epidemiological Studies Depression Scale compiled by Radloff (1977) and translated by Zhi-yan et al. (2009) has 20 items in total, including depressed affect (e.g., “I am bothered by something that was not bothering me recently.”), positive affect (e.g., “I feel no worse than others.”), somatic and retarded activity (e.g., “I don’t want to eat and have a bad appetite.”) and interpersonal (e.g., “I don’t think people are friendly to me.”) four dimensions (Zhi-yan et al., 2009). The scale uses 4 points, 0 means “no,” 3 represents “always,” and the four questions under the positive affect dimension are reverse scoring questions. Participants rated the frequency of occurrence of the symptom in the last week based on the item description. The average score of all items represents the depression level of adolescents, and the higher score means the higher the degree of depression. Previous studies have shown that the scale has good reliability and validity. The Cronbach’α coefficient of the scale in this study was 0.901. The confirmatory factor analysis showed that: χ^2^*/df* = 6.421, *RMSEA* = 0.065, *SRMR* = 0.045, *TLI* = 0.876, *CFI* = 0.891, indicating that the scale has good reliability and validity.

### Statistical Analyses

SPSS 22.0 software was used for descriptive statistics and correlation analysis. Mplus 8 software was used for structural equation models to establish multiple mediation model and multi-group analysis to test gender differences, and Bootstrap method of repeated sampling 1,000 times was used to test the mediation effect and estimate the confidence interval (Mackinnon et al., 2004). With respect to goodness of fit, two classes of indexes (i.e., statistical indicators reflecting the degree of fit between the hypothesized conceptual model and the empirical data) were adopted: Absolute and Relative Goodness-of-Fit Indices. The former included χ^2^/*df*, the root mean square error of approximation (*RMSEA*) and standardized root mean square residual (*SRMR*) (Schafer and Graham, 2002; Hair et al., 2011). The latter comprised comparative fit index (*CFI*) and Tucker-Lewis Coefficient (*TLI*). Thresholds for good model fit were: χ^2^/*df* > 3.0, *RMSEA* < 0.08, *SRMR* < 0.08, *CFI* > 0.90, *TLI* > 0.90 (Marsh and Hau, 1996; Schermelleh-Engel et al., 2003). Statistical significance test level α = 0.05, *p < 0.05, ^**^p < 0.01, ^***^p < 0.001.

### Common Method Biases

Since the questionnaires required for the study all require the participants to self-report, in order to control possible Common method biases, this study takes the following measures for process control: (1) Conduct the test collectively, read out the instructions uniformly, and emphasize that the research is only used for scientific research, and all information is absolutely confidential; (2) Emphasize that there is no right or wrong answer, and the participants only need to choose the one that they agree with or is relatively most suitable option for them and set up reverse scoring questions; (3) All questionnaires are distributed and withdrawn on the spot. In addition, statistical tests are performed using Harman’s One-factor Test. It was found that there were 11 eigenvalues greater than 1, which explained 55.297% of the variation, and that the first factor explained only 18.609% of the variance. Therefore, there is no serious common method deviation problem. At the same time, the control for effects of the unmeasured latent methods factor was used to test whether there was a serious common method biases in this study (Xiong et al., 2013). After adding the common method factor, the model fitting index is higher (*RMSEA* = 0.043, *SRMR* = 0.034, *CFI* = 0.982) than that without adding the common method factor (*RMSEA* = 0.070, *SRMR* = 0.045, *CFI* = 0.938), but they are all less than 0.05. It also shows that there is no serious common method biases problem.

## Results

### Describe Statistics and Related Analyses Between Variables

Correlation analysis shows that the correlation coefficient of the total score of each variable is between 0.095 and 0.447 (all *p* < 0.01). The differential test showed that the level of depression and mobile phone addiction was significantly higher in girls than in boys, and that the level of cyberbullying victimization and cyberbullying perpetration in boys was significantly higher than that in girls. See Table 1.

**TABLE 1:** Describes statistics and correlation analysis matrix of variables.

	Male		Female						
Variable	*M*	*SD*	*M*	*SD*	t	1	2	3	4
(1) DE	0.819	0.548	0.992	0.574	−5.389***	1			
(2) MPA	1.479	0.642	1.629	0.639	−4.128***	0.380**	1		
(3) CV	0.242	0.371	0.197	0.314	2.253*	0.241**	0.174**	1	
(4) CP	0.065	0.205	0.027	0.111	3.813***	0.095**	0.110**	0.447**	1

N = 1297, *p < 0.05, **p < 0.01, ***p < 0.001. MPA=mobile phone addiction; CV=cyberbullying victimization; CP=cyberbullying perpetration; DE=depression.

### Multiple Mediation Model

Test the measurement model before modeling (Wen and Ye, 2014), and build a structural equation model based on theoretical assumptions. In the present study, due to the complexity of the model and many estimated parameters, the item parceling strategies was used to simplify the model in the subsequent analysis. The two variables of cyberbullying victimization and preparation both satisfied the unidimensional and homogeneous conditions. The shortening method in the factorial algorithm was used to pack each variable into three indicators (Yan and ZhongLin, 2011). Mobile phone addiction included six dimensions: withdrawal behavior, salience behavior, social comfort, negative effects, app use of App, and app update renewal of App; and depression included depressed affect, positive affect, somatic and retarded activity and interpersonal four dimensions, which were multidimensional scales. Moreover, the internal-consistency approach was adopted for parceling, that is, the topics under the same dimension were packaged. This could reduce the differences within the group and increase the consistency of the indicators.

**FIGURE 2:**
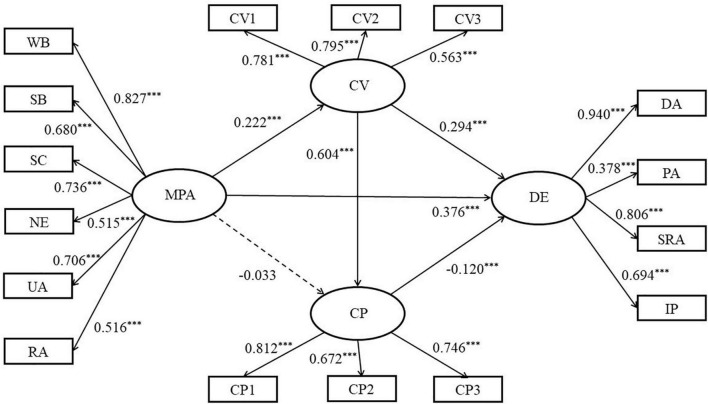
Multiple mediation model. Path values are the path coefficients (standard errors). ^***^Correlation is significant at the 0.001 level (2-tailed), MPA, mobile phone addiction, WB, withdrawal behavior, SB, salience behavior; SC, social comfort; NE, negative effects; UA, use of application (App); RA, renewal of app; CV, cyberbullying victimization; CP, cyberbullying perpetration; CV1–CV3 and CP1–CP3 are packaged dimensions; DE, depression; DA, depressed affect, PA, positive affect; SRA, somatic and retarded activity; IP, interpersonal.

The measurement model includes four latent variables of mobile phone addiction and depression, and 16 observation variables. Taking mobile phone addiction as an independent variable, depression as a dependent variable, cyberbullying victimization and cyberbullying perpetration as mediating variable, using structural equation model for path analysis, maximum likelihood method for parameter estimation, Bootstrap method (sample times is 1,000) to conduct a mediation effect test. The model is shown in Figure 2. Fit analysis showed that the data fit the model well: χ^2^/*df* = 4.984, *RMSEA* = 0.055, *SRMR* = 0.037, *TLI* = 0.938, *CFI* = 0.949. Mobile phone addiction significantly positively relates depression (β = 0.376, *p* < 0.001) and cyberbullying victimization (β = 0.222, *p* < 0.001), and has no significant relation on the cyberbullying perpetration (β = −0.033, *p* > 0.05). The cyberbullying victimization (β = 0.294, *p* < 0.001) and the cyberbullying perpetration (β = −0.120, *p* < 0.05) have a significant relation on depression, and the cyberbullying victimization has a significant negative relation on the cyberbullying perpetration (β = 0.604, *p* < 0.001).

A further examination of the mediation effect (Table 2) showed that the Bootstrap 95% confidence interval of the total indirect effect of cyberbullying victimization and cyberbullying perpetration between mobile phone addiction and depression was not included the value of 0. It shows that there are significant mediating effects in the effect of mobile phone addiction on depression. This mediating effect consists of three paths: First, the path (Indirect1) consisting of mobile phone addiction → cyberbullying victimization → depression. The confidence interval of its indirect effect does not contain a value of 0, indicating that the indirect effect produced by this path is significant (effect value is 0.068); second, the path (Indirect2) consisting of mobile phone addiction → cyberbullying perpetration → depression. The confidence interval of its indirect effect contains a value of 0, indicating that the indirect effect of this path has not reached a significant level; third, the path (Indirect3) consisting of mobile phone addiction → cyberbullying victimization → cyberbullying perpetration → depression. The confidence interval of its indirect effect does not contain a value of 0, indicating that the indirect effect produced by this path is significant (effect value is −0.027).

**TABLE 2:** Testing the pathways of the multiple mediation model.

			95% CI	
Path	Effect size	*SE*	Lower	Upper
Indirect 1	0.065	0.016	0.037	0.099
Indirect 2	0.004	0.004	–0.002	0.016
Indirect 3	–0.016	0.008	–0.035	–0.002
Total mediation effect	0.053	0.013	0.032	0.082
Direct	0.376	0.035	0.301	0.440

### Multi-Group Comparison of Mediation Model

In order to test whether the mediation model had cross-group stability, the present study conducted a multi-group path analysis on the gender difference of the model. The results showed that the model had statistically significant differences in gender (χ^2^*/df* = 2.297, *p* < 0.001); further analysis found that gender played a role in the path from cyberbullying victimization to cyberbullying perpetration. The path coefficients of the boys and girls from cyberbullying victimization to cyberbullying perpetration were 0.856 (*p* < 0.001) and 0.387 (*p* < 0.05), respectively, indicating that gender had a moderating effect on the path from cyberbullying victimization to cyberbullying perpetration. Compared with girls, boys were more likely to develop cyberbullying behaviors after experiencing cyberbullying.

## Discussion

Based on the General Aggressive Model of cyberbullying and the Frustration-Attack Hypothesis, the present research examines the Sequential mediating effect of the cyberbullying victimization and cyberbullying perpetration between mobile phone addiction and depression in high school students. The research focuses on the cyberbullying phenomenon of high school students in the context of Chinese society, which is a systematic empirical study of individual cyber bullying in mid-teens. On the one hand, it can fill the gaps in the current domestic cyberbullying empirical research to a certain extent. On the other hand, it can improve the influence mechanism of personal technology use traits on cyberbullying in the General Aggressive Model of cyberbullying and promote the development of existing cyberbullying research. The research conclusions help clarify the mechanism of cyberbullying in the mobile era, and provide a new perspective for the design and implementation of targeted interventions.

### Direct Impact

The results of this study show that H1 is established, high school students have positive predictive effects on depression, and high school students with more severe mobile phone addiction have higher levels of depression. Consistent with existing research findings (Çağan et al., 2014; Xiaodan et al., 2016; Alhassan et al., 2018), mobile phone addiction can lead to depression, anxiety, and sleep problems (Thomee, 2018), and teenagers who spend longer on mobile social media score higher on the depression scale on average (Calpbinici and Tas Arslan, 2019). It supports the research on mobile phone addiction and other psychosocial adaptation problems, and shows that mobile phone addiction is an important factor affecting the level of psychosocial adaptation of high school students. Mobile phone addiction can easily cause students to avoid learning tasks that need to be completed, reduce the efficiency of task completion, and have a negative impact on the quality of individual sleep (Thomee, 2018), accompanied by other negative symptoms such as high levels of anxiety, depression, negative self-esteem, physicalizing, and hostility (Bozzola et al., 2019; Calpbinici and Tas Arslan, 2019). Therefore, educators should pay attention to the depression of high school students addicted to mobile phones, carry out psychological guidance in a timely manner, and alleviate depression by guiding students to use mobile phones rationally.

### Mediating Role

The results of this study confirm the H2, that high school students’ mobile phone addiction positively predicts cyberbullying victimization, and cyberbullying victimization positively predicts depression; that is, cyberbullying victimization plays a mediating role between high school students’ mobile phone addiction and depression. Consistent with existing research, there is a positive correlation between cyberbullying and internet addiction (Tsimtsiou et al., 2017; Imek et al., 2019; Wachs et al., 2020). The findings support the General Aggressive Model of cyberbullying, in which mobile phone addiction, as a characteristic of personal technology use, will increase the possibility of being a victim of cyberbullying in high schools (Kowalski et al., 2014). On the one hand, as high school students who are addicted to mobile phones are more exposed to virtual Internet environments and thus receive more hostile messages on the Web (Alvarez-Garcia et al., 2015; HwaJin and Wanju, 2021). On the other hand, mobile phone-addicted teens exhibit higher levels of emotional problems and social dysfunction problems (Jain and Agrawal, 2020; Sheinov, 2021), is more likely to be targeted for cyber violence. Moreover, the anonymity of cyberbullying increases the negative impact of bullying victims by preventing them from judging the source of bullying, and non-face-to-face communication prevents victims from venting the negative emotions generated by bullying, thereby increasing their level of depression. Therefore, for high school students with high level of mobile phone addiction, educators should pay attention to the risk of their cyberbullying victimization, on the one hand, provide crisis intervention counseling for the victimization of cyberbullying, to prevent depression caused by the cyberbullying, and on the other hand, from the perspective of taking measures to alleviate mobile phone addiction to reduce the possibility of cyberbullying high school students.

### Sequential Mediating Effect

This study shows that the cyberbullying perpetration negatively predicts depression in high school students, while mobile phone addiction does not have a significant predictive effect on the cyberbullying perpetration, that is, the mediating effect of cyberbullying perpetration between mobile phone addiction and depression in high school students is not significant. What’s more, the cyberbullying victimization significantly positively predicts the cyberbullying perpetration, that is, the cyberbullying victimization and the cyberbullying perpetration have a sequential mediating effect between mobile phone addiction and depression in high school students. The conclusions of this study support the significant correlation between problematic mobile phone use and the cyberbullying victimization and the cyberbullying perpetration (Tsimtsiou et al., 2017; Gül et al., 2018), and there is a significantly positive correlation relationship between the cyberbullying victimization and the cyberbullying perpetration (Cañas et al., 2019). Contrary to the conclusions of existing research, this study shows that mobile phone addiction of high school students cannot directly increase their cyberbullying perpetration, but the victimization of cyberbullying caused by mobile phone addiction can trigger high school students to perpetrate cyberbullying behaviors and relieve their depression levels. The transition from victimized behavior to bullying behavior supports the Frustration-Attack Hypothesis that external attacks will cause the individual to suffer frustration and form a willingness to attack, and the cyberbullying information experienced serves as a weapon to initiate information. In addition, according to GAM, cyberbullying victims have severely depleted self-control resources and are more likely to make impulsive behaviors. Therefore, their willingness to bully can easily turn into aggressive behavior (Berkowitz et al., 2016). At the same time, the victim’s bullying of others has played a role in venting negative emotions to a certain extent, and due to the disinhibition effect brought by the anonymity of cyberbullying, the victim has a small exposure risk and psychological burden for harming others, thus showing more aggressiveness, and alleviating depression caused by self-victimization through cyberbullying of others.

### Multi-Group Comparison of Mediation Model

The research results confirmed Hypothesis H4, that is, compared with female high school students, boys are more likely to develop from cyberbullying victimization to cyberbullying perpetration. Consistent with existing research, boys’ cyberbullying victimization, and cyberbullying perpetration levels are higher than those of girls (Aricak, 2009; Erdur-Baker, 2010; Gonzalez-Cabrera et al., 2019; Martinez-Pecino and Duran, 2019), who is more likely to be attacked by cyberbullying. Although gender issues have always been controversial in previous research on cyberbullying (Barlett and Coyne, 2014), the results of this study support the claim that boys are more likely to be perpetrators of cyberbullying. This may be because boys are more likely to have the background and conditions for cyberbullying because they have access to the Internet and spend more time on the Internet every day (Zhehui et al., 2010). In addition, boys usually have a low level of rumination, a high level of moral excuse and a low sense of guilt. After being bullied, they tend to use distorted consequences and victim attribution to defend their unethical behavior, implement cyberbullying to retaliate against others and alleviate the impact of ruminating emotions (Cancan et al., 2019). Moreover, studies have shown that boys have a higher degree of moral disengagement than girls (Dongling and Meifang, 2013), and the use of moral disengagement strategies allows young people to cognitively moralize their unethical behaviors, so that boys are more likely to implement them without scruples. Cyberbullying (Wang et al., 2016). Other studies have shown that girls are better than boys in empathy (Jolliffe and Farrington, 2006), and individuals with low empathy are more likely to engage in cyberbullying (Kokkinos and Kipritsi, 2017), which also shows that boys are more likely to be cyberbullies than girls.

The findings of the present study explain the mechanism of transition of cyberbullying victims to bullying perpetrators, and although cyberbullying plays a role in alleviating negative emotions for individual victims, the end result is to promote the spread of cyberbullying, thus affecting the health and harmony of the entire network ecology. Therefore, in order to prevent the spread of cyberbullying behavior on the Internet, on the one hand, it is necessary to start from the source and use various interventions to alleviate the level of mobile phone addiction among high school students, thereby reducing the risk of cyberbullying victims and alleviating depression, and on the other hand, it is the key to prevent high school students from turning victims of cyberbullying into perpetrators and to build a healthy network by improving their self-control ability, coping with bullying experiences reasonably, and correctly diverting negative emotions.

## Limitations

In general, our outcomes were consistent with those of previous studies and fully verified their conclusions, reinforcing the authenticity and credibility of the present study. Nevertheless, the present study had several limitations which need to be supplemented by future research. Firstly, because all the studies are self-assessment data, but also a one-time collection, and most of the variables in this study are negative psychological behavior characteristics, the participants will take into account social praise and false deviations, and due to methodological limitations, these false deviations cannot be effectively separated. Therefore, future research can be combined with experimental design or follow-up methods to reduce the impact of these deviations on the study. In addition, in the mediating model, cyberbullying victimization and cyberbullying perpetration are only partially mediated in the relationship between mobile phone addiction and depression in high school students, indicating that there are other mediating variables affecting the relationship between the two, and future research should explore other possible intermediaries to explain the mechanisms by which high school students’ mobile phone addiction affects depression.

## Conclusion

This study is important for understanding the relationship between mobile phone addiction and depression among high school students, and shows that there is a link between cyberbullying victimization and cyberbullying perpetration in high school students’ mobile phone addiction and depression. In other words, high school students with a high level of mobile phone addiction have higher levels of depression and are likely to become victims of cyberbullying, and will alleviate their depression through cyberbullying others. Therefore, it is necessary to pay attention to the negative response measures after cyberbullying of high school students, especially boys, suffering from mobile phone addiction, guiding them correctly to cope with the crisis and alleviate negative emotions.

## Data Availability Statement

The original contributions presented in the study are included in the article/Supplementary Material, further inquiries can be directed to the corresponding author.

## Ethics Statement

The studies involving human participants were reviewed and approved by the questionnaire and methodology for this study was approved by the Human Research Ethics committee of the Kunming Medical University (Ethics approval number: 2021kmykdx6f66). Written informed consent to participate in this study was provided by the participants’ legal guardian/next of kin. Written informed consent was obtained from the individual(s), and minor(s)’ legal guardian/next of kin, for the publication of any potentially identifiable images or data included in this article.

## Author Contributions

YZ and YaC: conceptualization, methodology, and software. WW and ZG: data curation and writing original draft preparation. LY and YL: visualization and investigation. YoC and JL: supervision. RB: software and validation. HL: writing-reviewing and editing. All authors contributed to the article and approved the submitted version.

## Conflict of Interest

The authors declare that the research was conducted in the absence of any commercial or financial relationships that could be construed as a potential conflict of interest.

## Publisher’s Note

All claims expressed in this article are solely those of the authors and do not necessarily represent those of their affiliated organizations, or those of the publisher, the editors and the reviewers. Any product that may be evaluated in this article, or claim that may be made by its manufacturer, is not guaranteed or endorsed by the publisher.
